# Polydopamine Coated Nonspherical Magnetic Nanocluster for Synergistic Dual Magneto-Photothermal Cancer Therapy

**DOI:** 10.3390/polym17010085

**Published:** 2024-12-31

**Authors:** Gracia García-García, Marina Lázaro, Pedro Urquiza, Tania Romacho, Ángel V. Delgado, Guillermo R. Iglesias

**Affiliations:** 1 Department of Nursing, Physiotherapy and Medicine, University of Almería, 04120 Almería, Spain; tromacho@ual.es; 2 Chronic Complications Diabetes Lab (ChroCoDiL), University of Almería, 04120 Almería, Spain; 3 NanoMag Lab, Department of Applied Physics, Faculty of Science University of Granada, Planta-1, Edificio I+D Josefina Castro, Av. de Madrid, 28, 18012 Granada, Spain; marinalc@ugr.es (M.L.); adelagado@ugr.es (Á.V.D.); 4 Department of Applied Physics, School of Sciences, University of Granada, 18071 Granada, Spain; 5 Biosanitary Research Institute of Granada (ibs.GRANADA), University of Granada, 18001 Granada, Spain; 6 Biomedical Research Unit-Biotechnology Laboratory, Torrecárdenas University Hospital, C/Hermandad de Donantes de Sangre s/n, 04009 Almería, Spain; pu36@drexel.edu; 7 MNat Unit of Excellence, University of Granada, 18001 Granada, Spain

**Keywords:** dual magneto-photothermal cancer therapy, local hyperthermia, magnetic hyperthermia, nanocluster, nanoparticles, nanomedicine, magnetic nanoparticles, photothermal therapy, polydopamine, polymer

## Abstract

Local hyperthermia is gaining considerable interest due to its promising antitumor effects. In this context, dual magneto-photothermal cancer therapy holds great promise. For this purpose, the use of nanomaterials has been proposed. Therefore, the aim of this research is to develop a dual magneto-photothermal agent consisting of polydopamine-coated nonspherical magnetic nanoclusters. The physicochemical characterization of the nanoclusters was performed by electron microscopy, electron dispersive X-ray, dynamic light scattering, electrophoretic mobility, thermogravimetric analysis, and Fourier transform infrared spectroscopy. The biocompatibility of the nanoclusters was evaluated using human skin M1 fibroblasts. The potential of the nanoclusters as dual magneto-photothermal agents was investigated by applying an alternating magnetic field (18 kA/m and 165 kHz) and/or NIR laser (850 nm, 0.75 W/cm^2^). Nanoclusters showed a size of 350 nm consisting of nonspherical magnetic particles of 11 nm completely coated with polydopamine. In addition, they were superparamagnetic and did not significantly affect cell viability at concentrations below 200 µg/mL. Finally, the SAR values obtained for the nanoclusters demonstrated their suitability for magnetotherapy and phototherapy (71 and 41 W/g, respectively), with a synergistic effect when used together (176 W/g). Thus, this work has successfully developed polymeric-coated magnetic nanoclusters with the potential for dual magneto-photothermal cancer therapy.

## 1. Introduction

The exponential growth of nanomedicine has played a key role in the evolution of cancer therapies toward more effective and safer options [1]. As a result, some of the limitations of conventional cancer treatments (i.e., chemotherapy) are being overcome by the development of novel nanoparticle (NP)-based drugs [2]. In addition, novel therapeutic approaches such as antitumour hyperthermia have been satisfactorily translated into the clinic [3]. Although temperature rises above 40 °C were recognized as a potential antitumour strategy in the early 1970s [4], an efficient manner to induce it locally was lacking. The development of biocompatible NPs capable of acting as hyperthermia agents has been a breakthrough in controlling the thermal dose and developing targeted treatments [5].

Magnetic nanoparticles (MNPs) are well known for their use in magnetic hyperthermia therapy (MHT). MHT is based on the application of an alternating magnetic field (AMF) to magnetic NPs with superparamagnetic character, which leads to their behaviour as single dipoles that can be reoriented. The relaxation process (Brownian and/or Néel relaxation) leads to heat dissipation to their surroundings [6]. Photothermal therapy (PTT) is another hyperthermia modality mediated by MNPs that has also shown great potential both in vitro and in vivo [7,8,9] in the use of MNPs against cancer. In this case, the heating process occurs when near-infrared (NIR) light interacts with the movement of electrons on the surface of certain metallic NPs. Recently, the use of MNPs for dual magneto-photothermal therapeutic strategy has demonstrated superior antitumour effects compared to each modality applied independently [10]. Therefore, the development of MNPs for dual hyperthermia could enhance efficiency and, thus, increase the future clinical of antitumour therapies.

Surface modification of MNPs with biocompatible polymers is an attractive strategy for the development of multifunctional NPs to enhance therapeutic outcomes in cancer therapy [11,12,13,14]. Some of the reasons that support the use of polymers as coatings for MNPs are their ability to sustain and control drug release [11], improved biocompatibility profile, and colloidal stability [15,16]. Polydopamine (PDA) is a polymer recently proposed for biomedical applications [17,18]. PDA has emerged as a mussel-inspired biomaterial, as it has been found to be responsible for the strong adhesion of mussels to wood or stone under wet conditions of high shear stress from water flow [19]. Structurally, this polymer is also similar to melanin, a pigment capable of absorbing light to protect the skin [18]. Due to these two characteristics, its potential applications in nanomaterial surface modification and PTT are under investigation [20]. Furthermore, PDA is promising for controlled drug delivery due to its NIR-mediated heating potential [21] but also due to its reactive oxygen species-induced degradation [22].

In the present study, we develop a novel approach to enhance the local hyperthermia potential and, thus, develop a new generation of hyperthermia-based therapy against cancer. The strategy combines two aspects: a dual magneto-photothermal therapeutic strategy consisting of the simultaneous application of MHT and PTT and the design of a nanothermal agent with high potential in PTT and MHT, as well as a biocompatible profile. Herein, we report the development of PDA-coated magnetic nanoclusters (PDA-MNCs), together with a comprehensive chemical and physical characterization and the preliminary evaluation of their biocompatibility. Nonspherical MNPs were synthesized and clustered when dopamine molecules were polymerized to form a homogeneous coating. We have thoroughly evaluated the potential use of PDA-MNCs as dual magneto-photothermal agents. To the best of our knowledge, this is the first time that magnetic clustering has been performed using a PDA polymeric coating for simultaneous use in MHT and PTT. This strategy is promising because it could enhance the hyperthermia potential by the synergistic effect of both modalities (MHT and PTT) and/or both stimuli-responsive materials (PDA and MNPs). Hence, we believe that this novel nanostructure could be considered an example of a new generation of dual magneto-photothermal antitumor NPs.

## 2. Materials and Methods

### 2.1. Materials

Ammonium hydroxide solution, ferric acetylacetonate (99%), dopamine hydrochloride, phosphate buffer saline (PBS), glutaraldehyde, acetic acid, crystal violet solution (1%, aqueous solution) and antibiotic-antimycotic solution (10,000 units penicillin, 10 mg streptomycin and 25 μg amphotericin B per mL) were purchased from Merck KGaA (Darmstadt, Germany). Decanoic acid (99%) and dibenzyl ether (99%) were purchased from ACROS Organics™ (Leuven, Belgium). Fetal Calf Serum (FCS) and 0.05% trypsin-EDTA were supplied by GibcoTM (ThermoFisher, Barcelona, Spain). Potassium nitrate (KNO_3_, MW: 101.10 g/mol) was purchased from VWR International, LLC (Barcelona, Spain). Water was deionized and filtered with a Milli-Q Academic^®^, Millipore, Alsace, France. All chemicals were of analytical quality.

### 2.2. Methods

#### 2.2.1. Synthesis of Nonspherical Magnetic Nanoparticles

Nonspherical MNPs were synthesized using a decanoic acid-modified thermal decomposition methodology [23] with some modifications. The synthesis started with the addition of 0.35 g (1 mmol) of ferric acetylacetonate and 0.69 (4 mmol) of decanoic acid to 25 mL of dibenzyl ether. The resulting suspension was kept under a N_2_ atmosphere throughout the synthesis, and the heating required for the synthesis process was provided by a heating mantle (Electrothermal™ Stuart™ EM Series, Waltham, MA, USA). After degassing for 90 min, the reagents were heated at a rate of 12 ± 0.5 °C/min to 200 ± 2 °C, where the temperature was maintained for 2.5 h. The synthesis temperature was then increased again at a rate of 3 ± 0.5 °C/min until the reflux temperature of 280 ± 2 °C was reached. These conditions were maintained for 1 h, when the heating was removed, and the resulting products were cooled to room temperature. The resultant MNPs were centrifuged at 13,000 rpm for 20 min (Mikro 220R, Hettich Zentrifugen, Kirchlengern, Germany) and resuspended three times in ethanol before being stored at 4 °C.

#### 2.2.2. Synthesis of Polydopamine and Polydopamine-Coated Magnetic Nanoclusters

A classical Stöber method was used for the polymerization of dopamine, as previously described [24,25]. To obtain bare PDA NPs, 0.21 mL of ammonium hydroxide was mixed with a solvent consisting of 10 mL and 4.25 mL of ethanol and water, respectively. A 1.05 mL aqueous solution of 44.36 mg dopamine hydrochloride was then added dropwise at RT. Finally, the reagents were kept under mechanical stirring (500 rpm) for 96 h.

A PDA:MNPs ratio of 10:1 was chosen to obtain PDA-MNCs based on previous research [25]. In this procedure, 0.21 mL of ammonium hydroxide was mixed with 9.5 mL of water. A dispersion of 4.22 mL of ethanol containing 4.36 mg of MNPs was then added. Lastly, 1.05 mL aqueous solution of 44.36 mg dopamine hydrochloride was added dropwise at RT, and the reagents were kept under 500 rpm mechanical stirring for 96 h.

In both cases, the resulting NPs underwent repeated centrifugation cycles at 13,500 rpm for 10 min (Mikro 220R, Hettich Zentrifugen, Germany) and were resuspended in Milli-Q water until the supernatant conductivity was ≤10 μS/cm.

#### 2.2.3. Characterization of the Polydopamine-Coated Magnetic Nanoclusters

The size of MNPs and PDA-MNCs was assessed by electron microscopic analysis. MNPs were analysed by transmission electron microscopy (HRTEM), and the average size was determined by measuring at least 25 particles in different images using JImage 2.14.0 software (University of Wisconsin, Madison, WI, USA). In addition to HRTEM, PDA-MNCs were also studied by high-angle annular dark field scanning transmission electron microscopy (HAADF-STEM) (HAADF TALOS F200X, Thermofisher Scientific Inc., Waltham, MA, USA; accelerating voltage of 200 kV). Elemental analysis was performed during these electron microscopy observations [energy dispersive X-ray (EDX) spectrometer, Bruker Nano GmbH, Karlsruhe, Germany].

Dynamic light scattering (DLS) analysis was used to evaluate hydrodynamic size (and polydispersion or PdI), while the zeta potential (*ζ*) of the particles (*n* = 9) was measured by electrophoretic mobility. Samples measurements (0.1%, *w*/*v* NPs dispersion in water) were conducted at 25.0 ± 0.1 °C using a Zetasizer Nano-ZS, Malvern Instruments Ltd., Malvern, United Kingdom, at a scattering angle of 90°. PDA coating of MNCs was qualitatively evaluated by determining the influence of pH in the presence of 10^−3^ M KNO_3_ and the ionic strength fixed with KNO_3_ concentrations on the *ζ* values of PDA and magnetic PDA NPs. Prior to measurements, NPs were incubated within the dispersion media under mechanical stirring (Stuart rotator SB3, Lebanon, NJ, USA) for 12 h.

Colloidal stability of the magnetic PDA NPs and PDA-MNCs in Milli-Q water. It was investigated by the determination of *ζ* values and DLS analysis according to Equation (1), where *t*_1_ represents the time duration for assessment (1 month) [26].

Colloidal stability at *t*_1_ = NPs size at *t*_1_/initial NPs size (1)

Thermogravimetric analysis (TGA) of the samples of nonspherical magnetic PDA and PDA-MNCs was performed under a N_2_ flow of 50 mL/min at a heating rate of 10 °C/min (TGA-50H SHIMADZU, Tokyo, Japan).

The structure of PDA-MNCs was confirmed by Fourier transform infrared (FTIR) spectroscopy (FT/IR-6200 spectrometer JASCO, Waltham, MA, USA; resolution of 0.25 cm^−1^). For that purpose, the PDA-MNCs spectrum was compared to those obtained for nonspherical magnetic and PDA samples.

The magnetization cycle was obtained at (20.0 ± 0.5) °C in a vibrating sample magnetometer (PPMS DynaCool, Quantum Design, San Diego, CA, USA, maximum applied field ± 10.000 Oe).

#### 2.2.4. Thermal Measurements

**Figure 1:**
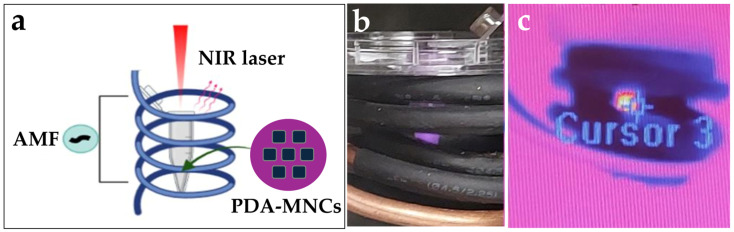
(**a**) Scheme of the dual therapy with simultaneous application of a near-infrared (NIR) laser and an alternating magnetic field (AMF) to the polydopamine-coated magnetic nanoclusters (PDA-MNCs), (**b**) image of the sample irradiated with the laser inside the coil, and (**c**) image of the thermal imaging camera used for the dual therapy.

The dual hyperthermia potential of the PDA-MNCs was evaluated by PTT and/or MHT capacity (Figure 1). For this purpose, Eppendorf tubes containing 100 μL of the sample dispersion in Milli-Q water were used at a concentration of 2.5 mg/mL. The temperature increase was monitored using a thermal imaging camera (FLIR E60, 320 × 240 pixel IR resolution, FLIR Systems, Inc., Waltham, MA, USA) in the middle of the Eppendorf tube.

For PTT testing, the setup consisted of an infrared laser (Laserland, Dresden, Germany, 850 nm, maximum power 1.6 W) pointing at the support holding the Eppendorf tube containing the sample. A PM160 Wireless Power Meter (Thorlabs GmbH, Münchner, Germany) was used to set the laser power. The MHT potential evaluation consisted of an AMF applied to the samples using a double four-turn coil 25.50 ± 0.05 mm in diameter made from 4.00 ± 0.05 mm copper tubing. A thermostatic bath was used to ensure that the heating capacity of the sample was generated solely by the NPs, and thus, no significant temperature changes were due to the Joule effect. Field strength and frequency measurements at the sample site were accurately set to 165 kHz and 18 kA/m using the strength and frequency of the AC magnetic probe (NanoScience Laboratories Ltd., Staffordshire, UK) with a sensitivity of 10 μT. For dual hyperthermia, a combined setup consisting of both infrared laser and AC magnetic field was applied simultaneously using the same methodology described for single measurements, as it has been illustrated in Figure 1.

The Specific Absorption Rate (SAR) values were calculated experimentally using Equation (2), as they represent the amount of heat transferred from the particles to their surroundings.

(2) $$SAR=\frac{C_{liq}\rho_{liq}}{\phi }\frac{dT}{dt}$$

where $C_{liq},\rho_{liq}$ are the mass-specific heat and the density of the suspension, and ϕ is the NPs concentration (*w*/*v*). The essential quantity is the rate of temperature increase dT/dt, which is determined by the linear model [27].

#### 2.2.5. Cell Culture

Human skin-derived M1 fibroblasts were cultured in Dulbecco’s modified Eagle’s medium (DMEM) pH 7.4, supplemented with 10% (*v*/*v*) FCS and antibiotic-antimycotic solution (0.2%) as previously described [28]. Cells were incubated at 37 °C with 5% CO_2_. Medium was changed every 48 h, unless stated. All reagents employed in the cytotoxicity assays were supplied by GibcoTM (ThermoFisher Scientific, Waltham, MA, USA).

#### 2.2.6. Crystal Violet Assay

A previously reported crystal violet staining protocol was followed to assess the potential cytotoxicity of PDA-MNCs [29]. Briefly, M1 cells were grown in a 96-well plate. After 24 h, the cells were exposed to UV and ethanol-sterilized NP samples. After 24 h of incubation, the medium was removed from each well by aspiration, and the cells were washed with sterile PBS. The cells were then fixed with glutaraldehyde (1%). After 15 min, the cells were washed with PBS 4X, and excess PBS was removed by decanting the plate. Crystal violet dye (0.1% *v*/*v*) was then added, incubated for 20 min, and repeatedly washed with deionized water until no colour leaked from the plate. Finally, the plate was dried overnight, and the dye was solubilized with 10% acetic acid. The absorbance of the remaining dye was measured in each well at 590 nm using a microplate reader spectrophotometer (INNO, LTek, Seongnam-si, Republic of Korea).

#### 2.2.7. Statistical Analysis

Statistical analysis was carried out using one-way ANOVA followed by Bonferroni correction to determine statistical significance (*p* ≤ 0.05). All statistical analysis was performed using GraphPad Prism (version 10.1.2, La Jolla, CA, USA).

## 3. Results and Discussion

### 3.1. Characterization

#### 3.1.1. Particle Geometry and Surface Properties

**Figure 2:**
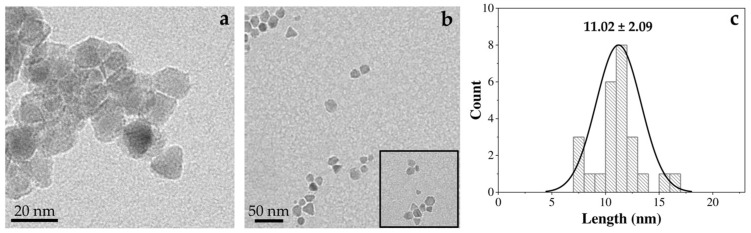
(**a**,**b**) HRTEM and (**c**) particle size distribution of the MNPs.

The synthesized MNPs were analyzed by HRTEM. The results in Figure 2a show that the particles obtained had a nonspherical shape. Furthermore, a high monodispersity is observed in Figure 2b. The anisotropy proper of nonspherical particles is an interesting feature for their application in hyperthermia due to their contribution to the consecution of high SAR values [6,10,23]. Figure 2c shows the appropriate mean size and polydispersity (11 ± 2 nm) (Figure 2c), associated with a safety profile for the in vivo administration [6].

**Figure 3:**
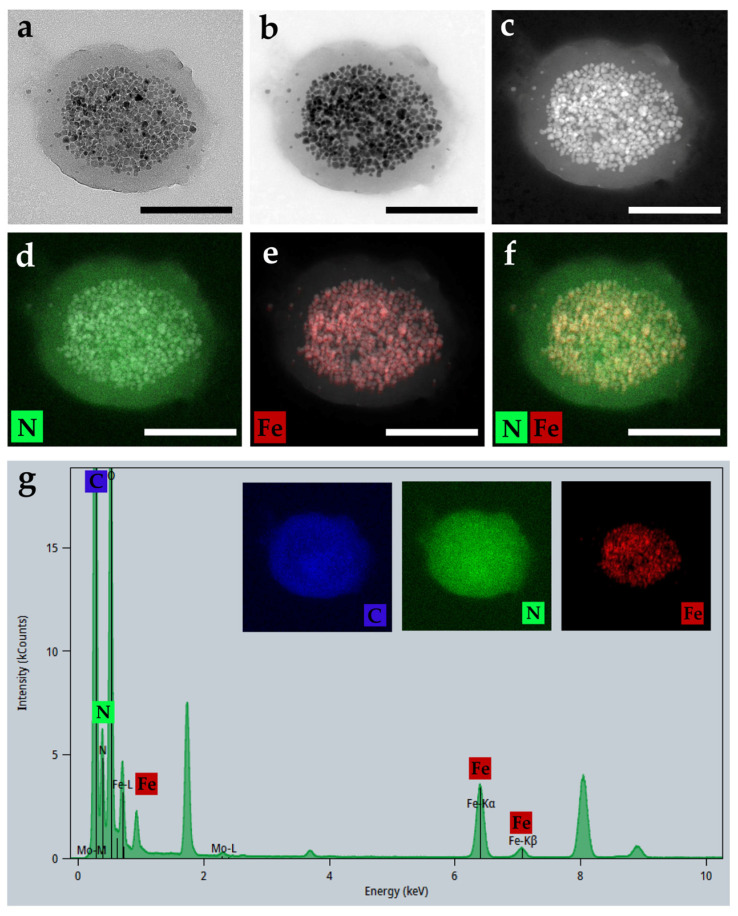
(**a**) High-resolution transmission electron microscopy (HRTEM), (**b**,**c**) high-angle annular dark field scanning transmission electron microscopy (HAADF-STEM), (**d**–**f**) energy dispersive X-ray (EDX) evaluation of the PDA-MNCs, and (**g**) EDX spectra and inserts of C,N and Fe element distributions. Bar length: 200 nm.

The structure of PDA-MNCs was assessed by HRTEM, HAADF-STEM, and EDX (Figure 3).

Figure 3 illustrates that PDA has completely coated several individual magnetic particles constituting a core. Specifically, Figure 3a–c show two different contrast regions, reflecting how the electrons interact more strongly in the core where the MNCs are located than in the coating due to the presence of heavier and lighter atoms, respectively. Then, Figure 3d–f confirmed the results by an elemental analysis of N and Fe. N element is present in PDA but not in MNPs. The opposite situation was encountered for Fe, proper from MNPs. Finally, a more specific EDX analysis was performed to understand the global composition and the proper surface and internal parts of the particles (Figure 3g). The EDX spectra show that the particles contain C, N, O, and Fe as the main elements. The images inserted in the EDX spectra show that C is present in both nanomaterials, and thus, it can be identified in the whole structure. The case of N and Fe is different as the N element is identified in PDA but not in MNPs, and the opposite situation was encountered for Fe, proper from MNPs.

DLS analysis of PDA-MNPs was performed to determine the average size. Such analysis was performed at the time of the particle obtention, resulting in (370 ± 30), but also after 1 month (470 ± 30). Therefore, the calculated colloidal stability using Equation (1) was 1.3 in water, which falls within the acceptable range of 1–1.5 according to the literature [30,31].

The electrophoretic surface properties were determined using the *ζ* evaluation of the PDA-MNCs as a qualitative assessment of the surface changes during the preparation process. The *ζ* measurement showed a similar trend for both PDA and PDA-MNC particles (Figure S1), demonstrating the similar chemical surface composition and, thus, confirming the PDA polymeric coating of the magnetic core previously identified in the TEM and EDX analysis (see Section 3.1.1). These particles exhibited negative values in most of the media pH and all the ionic strengths studied (Figure S1). It is likely that the predominance of catechol units (acidic OH groups) over aminoethyl groups in PDA is responsible for these results [32]. It is exclusively in a dispersion medium with an important concentration of H^+^ (pH 3) that the negative charges of the OH groups are neutralized, resulting in a positive charge of the particles.

#### 3.1.2. Infrared Analysis

**Figure 4:**
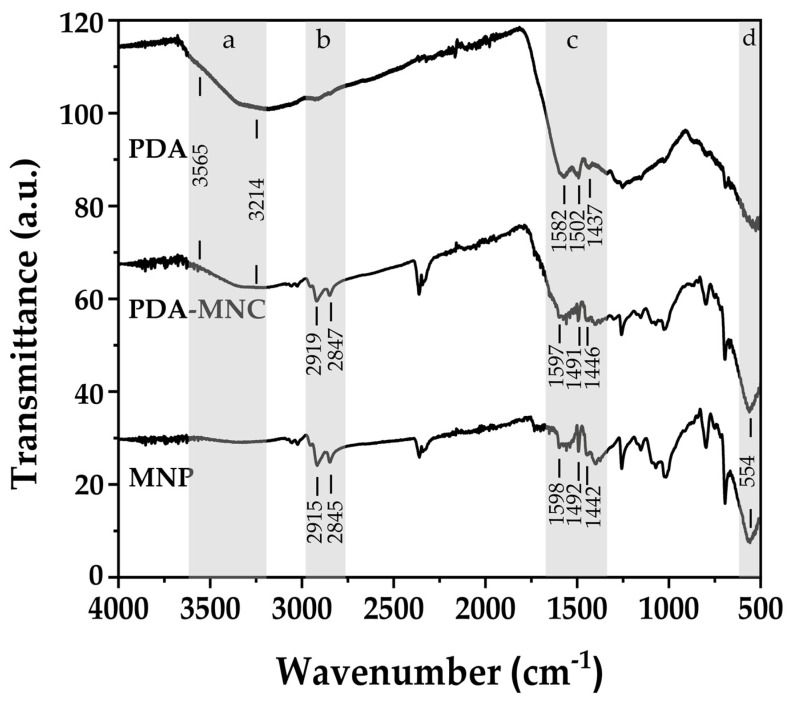
FTIR analysis of the particles studied where the most significant peaks for comparison between MNP, PDA, and PDA-MNC have been identified in the highlighted regions (**a**–**d**).

The PDA-MNCs were characterized using FTIR, comparing them with PDA and MNP samples (Figure 4). FTIR spectrum of synthetic PDA nanoparticles revealed a large peak spanning around 3600–3200 cm^−1^, consistent with the presence of a hydroxyl structure as well as water (Figure 4a). In contrast with dopamine, polydopamine is characterized by a much broader peak in that region [33]. That feature confirms the polymerization process of dopamine in both PDA and PDA-MNC particles obtained. Asymmetric and symmetric C-H- stretching vibrations from the decanoic acid coating of iron oxide particles in MNP and PDA-MNC correspond to approximately 2915 and 2840 cm^−1^ peaks (Figure 4b) [34]. MNP and PDA-MNC spectra show peaks between 1505 and 1380 cm^−1^ range (Figure 4c), indicating an interaction of iron oxide particles with fatty acid in MNPs [34]. Also close to that region, PDA and PDA-MNC samples show peaks consistent with indole or indoline structures (N-H and catechol –OH; 1515 and 1605 cm^−1^ [33,35]. MNP and PDA-MNC spectra show 634 and 565 cm^−1^ peaks, characteristic of Fe-O lattice vibration (Figure 4d) [11,36].

#### 3.1.3. Thermogravimetric Analysis

**Figure 5:**
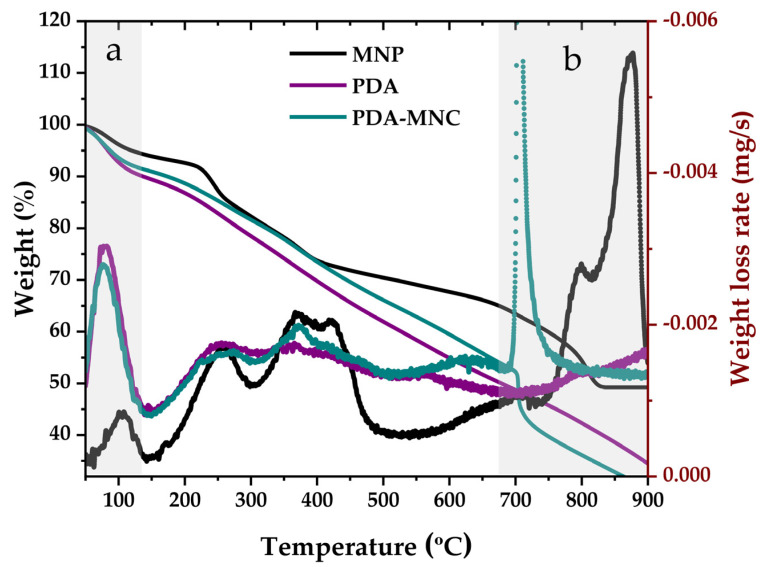
TGA spectra result for MNP, PDA, and PDA-MNC particles in terms of weight % and the obtained weight loss rate curve (mg/s). The most significant changes in the characterization of PDA-MNCs have been highlighted and identified as (**a**,**b**).

PDA and PDA-MNC TGA analysis demonstrated the effectiveness of the polymerization process, resulting in an undefined multi-step degradation profile (Figure 5) similar to previously reported PDA particles and in contrast to that of dopamine molecules [37,38].

The moisture peak of PDA-MNCs is significantly larger than that of MNPs (Figure 5a). This feature is also observed for PDA-NPs and highlights their hydrophilic character in addition to its presence in the PDA-MNCs. On the contrary, for hydrophobic MNPs, low moisture is obtained due to their decanoic acid surface composition (Figure 5a). The presence of MNPs in PDA-MNCs can be confirmed by the weight loss observed at temperatures above 650 °C, where magnetic materials degrade [36] (Figure 5b).

#### 3.1.4. Magnetic Behaviour

Magnetic characterization results of PDA-MNPs are depicted in Figure S2. The increasing and decreasing field ramps are almost coincidental. The low magnetic hysteresis of the composites suggests a potential application of the PDA-MNCs particles for biomedical purposes, given their moderate magnetization and their expectedly negligible risk of aggregation in blood vessels due to magnetic forces [39]. PDA-MNCs presented significantly lower saturation magnetization compared to bare MNPs. As previously reported by Guardia P. et al. [23], MNPs contained not only magnetite but also maghemite, goethite, and decanoic acid, resulting in a lower magnetic character than bare magnetite. Then, the results of the PDA-MNCs could be a contribution of the already reduced MNP saturation magnetization with the addition of the PDA content in the total mass, which resulted in a magnetic character similar to that previously reported for polymeric-coated MNPs [15,16].

### 3.2. Thermal Measurements

The experimental setting (Figure 1) was chosen to consider a potential clinical application. Therefore, MHT [40] and PTT [41] conditions where the effect of tissue damage caused directly by the AMF or laser power irradiation would be negligible were used.

**Figure 6:**
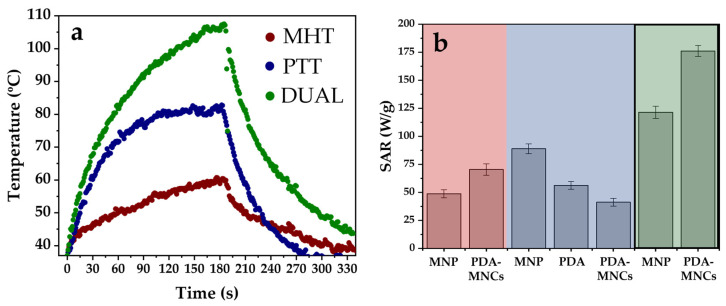
Temperature increases (**a**,**b**) SAR values for PDA-MNC dispersion when applying MHT with 165 kHz and 18 kA/m (Red range), PTT with 850 nm and 0.75 W/cm^2^ laser (blue range), and combined MHT and PTT (green range).

The PTT potential of NPs corresponds to their NIR optical absorption capacity. Both iron oxide colloids (specifically nonspherical magnetite) and PDA NPs exhibited significant NIR absorption and were shown to be suitable as PTT agents (Figure 6b) [10,42]. Then, we tested PDA-MNCs containing both nanomaterials under a NIR laser and observed that a high-temperature increase was obtained (Figure 6a). SAR values found for PDA-MNCs at 0.75 W/cm^2^ were more similar to those found for PDA obtained when compared to MNPs. This could be explained by the large amount of PDA forming a thick coating on the MNPs (Figure 3).

For the MHT modality, only MNPs are potential agents due to their superparamagnetic character (Figure S2). When a magnetic field was applied, a great temperature increase was obtained for PDA-MNCs (Figure 6a), which can be attributed to the MNPs’ presence and their cluster conformation within the PDA nanomatrix [36,43,44]. In fact, more efficient heating was achieved for PDA-MNCs than for MNPs, highlighting the importance of the multicore structure for magnetothermal performance (Figure 6b).

As mentioned, dual therapy refers to the situation in which an AC magnetic field is applied simultaneously to the laser irradiation with the aim of reaching a given therapeutic response while using a moderate magnetic field and laser stimuli. Results of the current work determine that the combination could enhance therapeutical outcomes, as a higher temperature increase was obtained for PDA-MNCs when compared to the single hyperthermia modalities (Figure 6a). This is an important result confirmed by SAR values (Figure 6b), which opens a way of optimizing the treatment while minimizing unwanted side effects from the simultaneous application of MHT and PTT, as it has been previously studied and described in our laboratory [29]. Furthermore, these findings highlight that the optimization of antitumor treatment based on hyperthermia could also be achieved by the combination of stimuli-responsive materials (i.e., PDA and MNPs) in a polymeric-coated-magnetic particle cluster.

### 3.3. Cell Culture and Cytotoxicity Assay

**Figure 7:**
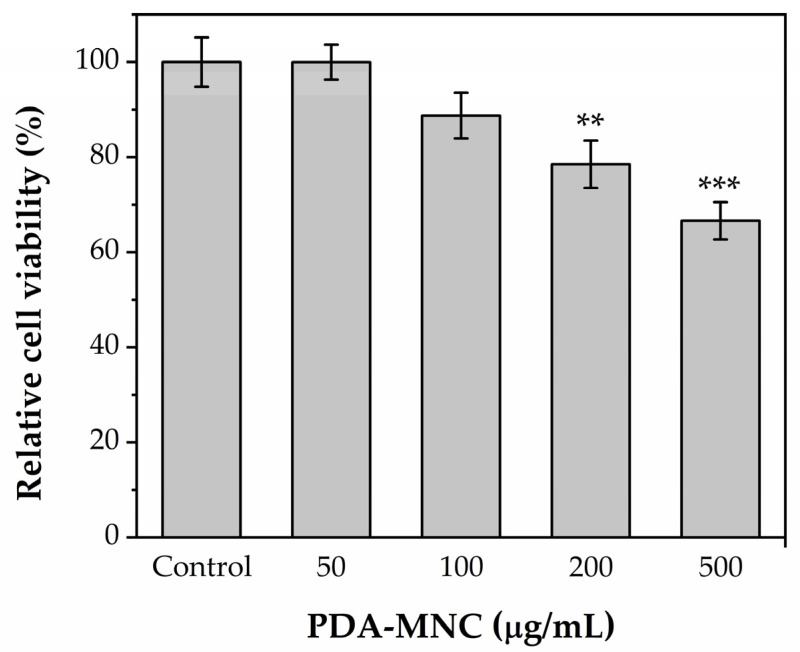
Effects of PDA-MNCs on M1 human skin fibroblast viability. Cell proliferation was tested after 24 h exposure to PDA-MNCs (0–500 μg/mL). Data are shown as the mean ± SEM of at least three independent experiments and expressed as a percentage of untreated control levels. Cytotoxicity assays were performed in triplicates under experimental conditions. Statistical significance was established at *p* values below or equal to 0.05; ** *p* ≤ 0.01; *** *p* ≤ 0.001.

Potential translation to the clinic of the engineered PDA-MNCs as therapeutic agents in antitumor hyperthermia strongly depends on the safety administration of the particles. Thus, a preliminary biocompatibility assay was performed using human fibroblasts, which provide a representative model of human tissue response. Specifically, we evaluated the biocompatibility of the designed nanomaterial, i.e., PDA-MNCs, by measuring the viability of human fibroblast-derived M1 cells incubated for 24 h with NP concentrations up to 500 μg/mL (Figure 7). No significant reduction in cell viability was observed at concentrations below 200 μg mL^−1^. Meanwhile, cell viability decreased significantly for 200 and 500 μg/mL. The results of this standard viability assay based on human fibroblast-derived M1 cells indicated the biocompatibility of the PDA-MNCs NPs with healthy tissue cells, which was consistent with previous findings [45,46].

## 4. Conclusions

This study presents the engineering of magnetic nanoparticle (MNP) clusters, controlled by the surface polymerization of polydopamine (PDA), for potential application in dual magneto-photothermal cancer therapy. The MNPs are synthesized using a decanoic acid-modified thermal decomposition methodology, which imparts a nonspherical shape and superparamagnetic behavior. These characteristics ensure both safety and effective performance in biomedical applications, such as antitumor hyperthermia therapy, MRI diagnostics, and magnetic targeting. To modify the hydrophobic decanoic acid coating and enable a controlled aggregation process, a hydrophilic, biocompatible, and stimuli-responsive polymer inspired by mussel adhesive properties, PDA, is employed as a coating material. The resulting PDA-coated magnetic nanoclusters (PDA-MNCs) exhibit preliminary biocompatibility at concentrations suitable for biomedical use and demonstrate a magneto- and photothermal response under alternating magnetic fields (AMF) and near-infrared (NIR) laser irradiation, respectively, generating localized heating. This research exploits the hyperthermia phenomenon induced by these stimuli, either individually or simultaneously, to propose a promising antitumor therapy approach: dual magneto-photothermal cancer therapy. The PDA-MNCs show an enhanced response to magnetic hyperthermia therapy (MHT) and photothermal therapy (PTT) due to the combination of the nanomaterial properties and the structured nanocluster design. Notably, the hyperthermia response is significantly amplified when MHT and PTT are applied simultaneously. The engineered polymer-coated magnetic nanoclusters offer a compelling platform for antitumor hyperthermia, providing a synergistic response in dual magneto-photothermal cancer therapy. These results are achieved under moderate AMF and NIR laser conditions, maintaining biocompatibility and demonstrating their potential for safe and efficient biomedical applications.

## Data Availability

Data is contained within the article or Supplementary Materials. The original contributions presented in this study are included in the article/Supplementary Materials. Further inquiries can be directed to the corresponding author(s).

## Supplementary Materials

The following supporting information can be downloaded at https://www.mdpi.com/article/10.3390/polym17010085/s1. Figure S1. Zeta potential (*ζ*, mV) of the PDA and PDA-MNC particles as a function of (a) pH and (b) the KNO_3_ molar concentration. Figure S2. Magnetization curves of the obtained PDA-MNCs performed at 300 K.
