# Supplementary material for: Polydopamine Coated Nonspherical Magnetic Nanocluster for Synergistic Dual Magneto-Photothermal Cancer Therapy
Source: Polymers (Basel). 2024 Dec 31;17(1):85. doi: 10.3390/polym17010085 (PMC11723388; doi:10.3390/polym17010085)
Supplement: Supplementary file 1 [file polymers-17-00085-s001.zip › polymers-3368958-supplementary.pdf]

Article

Polydopamine Coated Nonspherical Magnetic Nanocluster for Synergistic Dual Magneto-Photothermal Cancer Therapy

Gracia García-García [1,2,3,*], Marina Lázaro [3,4,5], Pedro Urquiza [6], Tania Romacho [1,2], Ángel V. Delgado [3,4,5,7] and Guillermo R. Iglesias [3,4,5,7,*]

1 Department of Nursing, Physiotherapy and Medicine, University of Almería, 04120 Almería, Spain; tromacho@ual.es
2 Chronic Complications Diabetes Lab (ChroCoDiL), University of Almería, 04120 Almería, Spain
3 NanoMag Lab, Department of Applied Physics, Faculty of Science University of Granada, Planta-1, Edificio I+D Josefina Castro, Av. de Madrid, 28, 18012 Granada, Spain; marinalc@ugr.es (M.L.); adelagado@ugr.es (Á.V.D.)
4 Department of Applied Physics, School of Sciences, University of Granada, 18071 Granada, Spain
5 Biosanitary Research Institute of Granada (ibs.GRANADA), University of Granada, 18001 Granada, Spain
6 Torrecárdenas University Hospital, Biomedical Research Unit-Biotechnology Laboratory, C/Hermandad de Donantes de Sangre s/n, 04009 Almería, Spain; pu36@drexel.edu
7 MNat Unit of Excellence, University of Granada, 18001 Granada, Spain
* Correspondence: graciagg@ual.es (G.G.-G.); iglesias@ugr.es (G.R.I.)

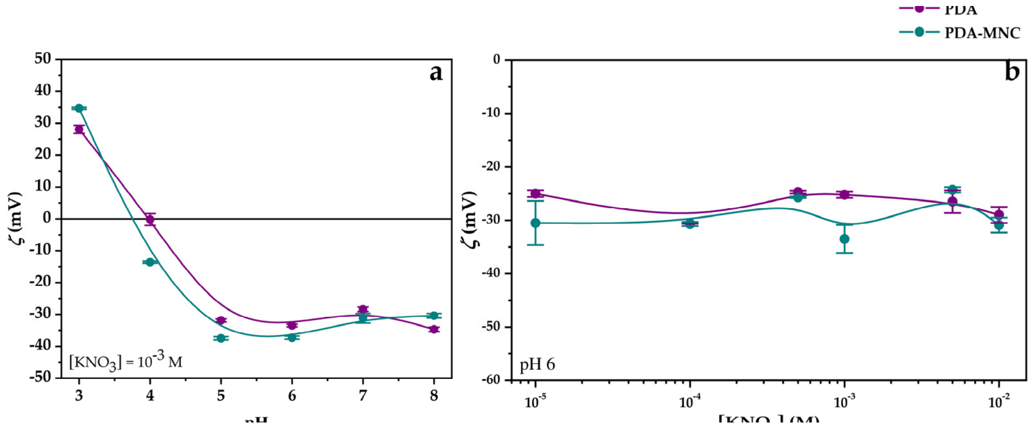

Figure S1. Zeta potential (ζ, mV) of the PDA and PDA-MNC particles as a function of (**a**) pH and (**b**) the KNO_3 molar concentration.

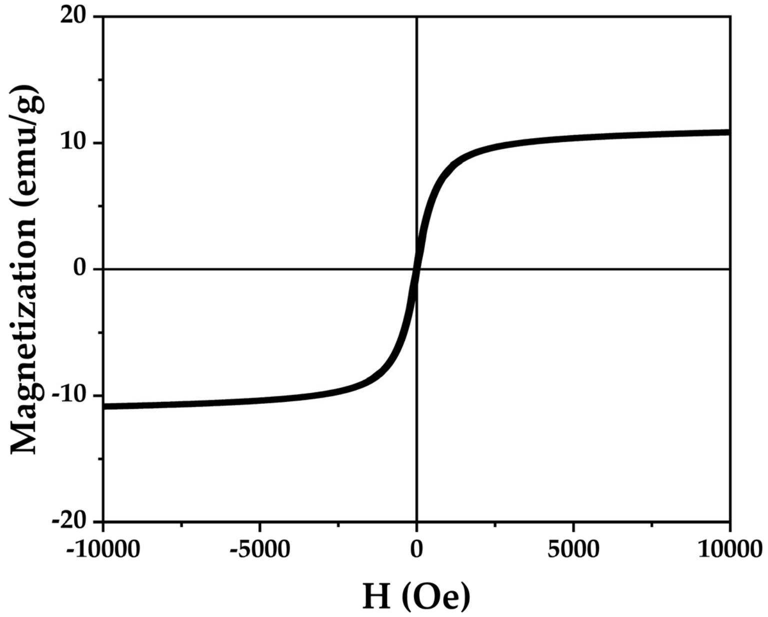

Figure S2. Magnetization curves of the obtained PDA-MNCs performed at 300 K.
